# Diagnostic extended usefulness of RMI: comparison of four risk of malignancy index in preoperative differentiation of borderline ovarian tumors and benign ovarian tumors

**DOI:** 10.1186/s13048-019-0568-3

**Published:** 2019-09-16

**Authors:** Shuang Zhang, Shan Yu, Wenying Hou, Xiaoying Li, Chunping Ning, Yingnan Wu, Feng Zhang, Yu Fei Jiao, Leo Tsz On Lee, Litao Sun

**Affiliations:** 1 Department of Ultrasound, The Secondary Affiliated Hospital of Harbin Medical University, Harbin, China; 2 Centre of Reproduction Development and Aging, Faculty of Health Sciences, University of Macau, Macau SAR, China; 3 Department of Ultrasound, The Affiliated Hospital of Qingdao University, Qingdao, China; 4 Department of Pathology, The Secondary Affiliated Hospital of Harbin Medical University, Harbin, China; 5 Department of Ultrasound, XuanWu Hospital of Capital Medical University, Beijing, China

**Keywords:** Borderline ovarian tumor, Preoperative evaluation, Risk of malignancy index, Differential diagnosis, Benign ovarian tumor

## Abstract

**Background:**

This study aimed to examine the performance of the four risk of malignancy index (RMI) in discriminating borderline ovarian tumors (BOTs) and benign ovarian masses in daily clinical practice.

**Methods:**

A total of 162 women with BOTs and 379 women with benign ovarian tumors diagnosed at the Second Affiliated Hospital of Harbin Medical University from January 2012 to December 2016 were enrolled in this retrospective study. Also, we classified these patients into serous borderline ovarian tumor (SBOT) and mucinous borderline ovarian tumor (MBOT) subgroup. Preoperative ultrasound findings, cancer antigen 125 (CA125) and menopausal status were reviewed. The area under the curve (AUC) of receiver operator characteristic curves (ROC) and performance indices of RMI I, RMI II, RMI III and RMI IV were calculated and compared for discrimination between benign ovarian tumors and BOTs.

**Results:**

RMI I had the highest AUC (0.825, 95% CI: 0.790–0.856) among the four RMIs in BOTs group. Similar results were found in SBOT (0.839, 95% CI: 0.804–0.871) and MBOT (0.791, 95% CI: 0.749–0.829) subgroups. RMI I had the highest specificity among the BOTs group (87.6, 95% CI: 83.9–90.7%), SBOT (87.6, 95% CI: 83.9–90.7%) and MBOT group (87.6, 95% CI: 83.9–90.7%). RMI II scored the highest overall in terms of sensitivity among the BOTs group (69.75, 95% CI: 62.1–76.7%), SBOT (74.34, 95% CI: 65.3–82.1%) and MBOT (59.18, 95% CI: 44.2–73.0%) group.

**Conclusion:**

Compared to other RMIs, RMI I was the best-performed method for differentiation of BOTs from benign ovarian tumors. At the same time, RMI I also performed best in the discrimination SBOT from benign ovarian tumors.

## Background

The concept and treatment of borderline ovarian tumors are in controversial for more than a century. Borderline ovarian tumors (BOTs) could form a separate entity that different with benign and malignant ovarian neoplasms. These tumors are histopathologically different by abnormal epithelium and may become cancer. Hence it is also called “ovarian low malignant potential tumor”, as those tumors are believed to have characteristics related to invasive ovarian cancer [1]. It was first described by Taylor in 1929 and officially classified by the International Federation of Gynecology and Obstetrics (FIGO) in 1971 and World Health Organization (WHO) in 1973 [2–4]. These tumors account for approximately 10–20% of all ovarian epithelial tumors, especially in women of reproductive age [1, 5]. So far, six subtypes of BOTs are identified as: serous (50–55%), mucinous (30–45%), endometrioid, clear cell, seromucinous and borderline Brenner tumor of the ovary [6].

Current findings suggested that the serous borderline ovarian tumors (SBOTs) have more potential to develop into low-grade serous carcinoma, while other borderline ovarian tumors present relative “inert” behavior [7]. Based on this conception, grouping BOTs into different histological subtype and distinction from benign ovarian tumors is of great translational research interests. The distinction of borderline from benign is important since the recommended surgery method is completely different, besides conservative fertility treatment [8]. As lacking effective indicators for preoperative diagnosis and with economic considerations, clinicians would not decide to send samples for an intraoperative frozen section examination if the tumor looks like “Benign” before the operation, which could make the clinical situation into a dilemma for a secondary surgery.

As BOTs have less distinct ultrasound characteristics, other preoperative examinations such as magnetic resonance imaging (MRI), computed tomography (CT), serum levels of CA125, CA199, and even biopsy are often not easy for a definitive diagnosis respectively [9–15]. However, precise preoperative evaluation of ovarian masses is important to decrease unnecessary anxiety and enable decisions for optimal treatment, especially for patients who wish to preserve their reproductive capacity and do not wish to take a secondary surgery. Thus, specific and sensitive methods for preoperation diagnosing ovarian borderline tumors are needed.

So far, there are only a couple of reports about evaluating the effectiveness of methods in the distinction between BOTs and benign ovarian tumors [16–18]. The risk of malignancy index (RMI) is probably the most commonly accepted and easy model [19]. RMI is an algorithm based on scores derived from ultrasound variables, menopausal status, and serum CA125 level. Till now, four versions, RMI I, II, III, and IV have been established and generally accepted by clinicians to distinguish malignant ovarian tumors from benign ones.

Our study was purposed to evaluate the availability and performance characteristics of the four RMIs to discriminate BOTs from benign ovarian tumors. Also, we are trying to provide an effective preoperational evaluation module between benign and borderline ovarian tumors in histological subgroups in order to facilitate clinicians choosing a best therapeutic strategy for patients.

## Materials and methods

### Patient clinical data

The clinical data of 912 women who underwent surgery for an ovarian mass in the Obstetrics and Gynecology Department, Second Affiliated Hospital of Harbin Medical University from January 2012 to December 2016 were obtained into our retrospective analysis. All subjects agreed with the ethics examination and signed informed consent. Only serous and mucinous borderline ovarian tumors (MBOTs) and benign ovarian tumors with complete laboratory data and definitive pathology report were included in this study. Moreover, the ultrasound parameters must be able to be extracted from patients in hospital records. All others were excluded. This study only accepts the final surgical pathology reports approved by two individual pathologists with consensus.

### Ultrasound examination

The ultrasound was performed transvaginally by Voluson E8 (GE Healthcare, Wauwatosa, WI, USA) with a 5- to 9-MHz transvaginal transducer. Patients lay in the lithotomy position after emptying the bladder. On condition that a mass was found to be too large to be observed completely transvaginally, a transabdominal repeat examination with a full bladder in the supine position was obtained using Voluson E8 with a 4- to 8-MHz transabdominal probe. The ultrasound characters and single greatest diameter of the tumor were recorded. If the ovarian masses were more than one, only the one with most complex morphologic characteristics was considered for statistical analysis. Visceral organs and peritoneal surfaces, including the omentum majus and lymph nodes surrounding the abdominal aorta and iliac arteries, were examined.

### RMI

**Fig. 1:**
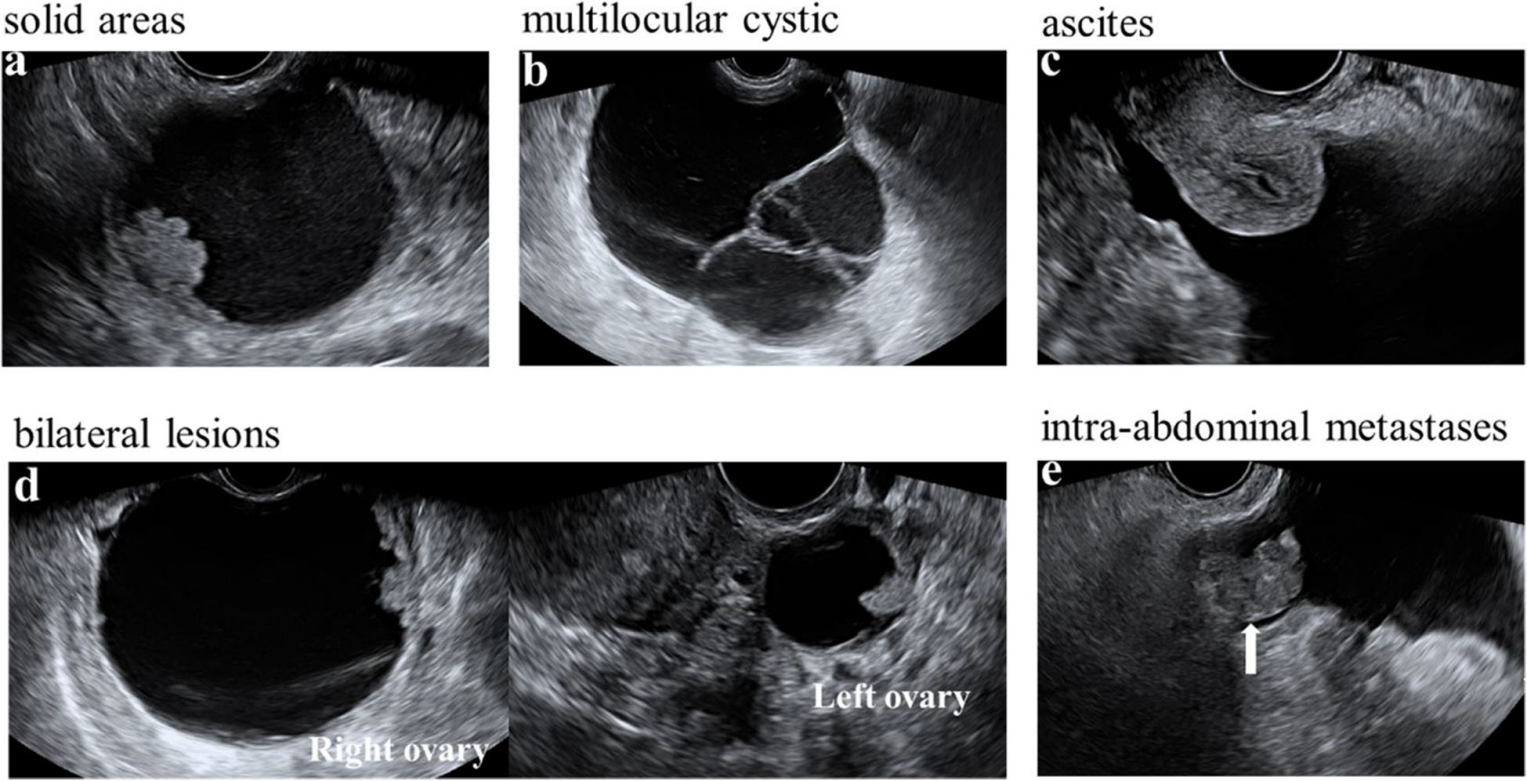
Illustrative ultrasound figures in RMI score algorithms. Each of the five ultrasound characters counts one point. **a** Unilocular SBOT with solid projection on the wall. **b** Multilocular MBOT. **c** Massive ascites. **d** Bilateral BOT lesions of one patient. **e** The arrow refers to the intra-abdominal metastase

Taken all data together, RMI I, RMI II, RMI III, and RMI IV were calculated for all qualified patients (Score algorithms in Table 1). Briefly, each of the ultrasound characters (multilocular cystic lesion, solid areas, bilateral lesions, ascites, intra-abdominal metastases findings in Fig. 1) is counting as one point. The final ultrasound score (U) was summed for each patient. Tumor size (S) was also recorded by ultrasound. The postmenopausal status was determined as age over 50 and amenorrhea for over 1 year, while all others were considered premenopausal. Serum CA125 value was extracted from laboratory test with the protocol provided by manufactory (ARCHITECT CA125 II Reagent Kit 2 K45, ARCHITECT i4000 immunoassay analyzer, Abbott, U.S.A.) and applied to each algorithm.

**Table 1:** Schematic presentation of four different RMI score algorithms

Variants	Ultrasound Score (U)^a^	Menopausal Score (M)	Tumor Size (S), cm^b^
RMI I = U × M × CA-125	U = 0 (0 parameter)	M = 1 (premenopausal)	Not applicable
	U = 1 (1 parameter)	M = 3 (postmenopausal)	
	U = 3 (≥2 parameters)		
RMI II = U × M × CA-125	U = 1 (0 or 1 parameter)	M = 1 (pre-menopausal)	Not applicable
	U = 4 (≥2 parameters)	M = 4 (postmenopausal)	
RMI III = U × M × CA-125	U = 1 (0 or 1 parameter)	M = 1 (premenopausal)	Not applicable
	U = 3 (≥2 parameters)	M = 3 (postmenopausal)	
RMI IV = U × M × S × CA-125	U = 1 (0 or 1 parameter)	M = 1 (premenopausal)	S = 1 (< 7)
	U = 4 (≥2 parameters)	M = 4 (postmenopausal)	S = 2 (≥7)

^a^Parameters: presence of a multilocular cystic lesion, solid areas, a bilateral lesion, ascites, and intra-abdominal metastasis

^b^Single greatest diameter

### Statistical analysis

All statistical analyses were performed by the SPSS ver. 20 (SPSS Inc., Chicago, IL, USA) and MedCalc ver. 15.8 (MedCalc Software, Mariakerke, Belgium). The Chi-square test was used to test differences in menopausal status, ultrasound score and tumor size. The Mann­Whitney U­test was applied when testing differences in the distribution of CA­125. Age was compared with the use of the Student’s t-test according to their distribution. ROC curves were constructed and the Area under the receiver operator characteristic curves (AUC) with binomial exact 95% confidence intervals were calculated between benign ovarian tumors and BOTs [20]. The diagnostic performance of the models was also expressed as sensitivity, specificity and positive and negative likelihood ratios. The method as previously described was used to calculate the difference between two AUCs [21]. Exact McNemar test was used to compare the sensitivity of the RMI I, RMI II, RMI III and RMI IV. Finally, synthetical evaluation of the diagnostic performance was measured by AUC, sensitivity, and specificity. The *p*-value < 0.05 was considered to indicate the statistically significant difference.

## Results

### Patient and tumor characteristics

In total, 541 cases (59.32%, 541/912) were qualified our criterion and included in our study. The histopathological classification of all cases (162 women with BOTs and 379 women with benign ovarian masses) is listed in Table 2. The majority of benign ovarian masses were mucinous cystadenoma (*n* = 96) and serous cystadenoma (*n* = 88). Histopathological results confirmed 113 SBOTs and 49 MBOTs. There was no significant difference in age and menopausal status among the BOTs group, SBOT and MBOT subgroup and benign group (*p* > 0.05). The difference was found statistically significant in the value of CA125 serum level and ultrasound score between the BOTs group, SBOT and MBOT subgroup and benign group (*p* < 0.05). For the tumor size, the *p* was < 0.05 between BOTs, MBOT group, and benign group. There was no significant difference in tumor size between SBOT and benign group (*p* = 0.505). Those clinical data above was summarized and illustrated in Table 3.

**Table 2:** Distribution of histopathologic diagnoses

Histological diagnosis	n	%
Benign (*n* = 379)		
Mucinous cystadenomas	96	25
Serous cystadenomas	88	23
Endometriotic cysts	79	20
Teratoma	60	16
Simple cysts	25	7
Theca fibroma	25	7
Brenner tumor	6	2
BOTs (*n* = 162)		
Borderline Serous cystadenoma	113	70
Borderline Mucinous cystadenoma	49	30

**Table 3:** The distribution of benign ovarian tumors and BOTs including subgroup of BOTs by age, menopausal status, ultrasound score, serum CA125, and tumor size

Variables	Benign (*n* = 379)	BOTs (*n* = 162)	*P* value	SBOT	*P* value	MBOT	*P* value
Age (years) Mean ± SD	37.73 ± 14.61	40.3 ± 15.12	0.065^b^	39.88 ± 14.32	0.168^b^	41.24 ± 16.92	0.12^b^
Menopausal status			0.88^c^		0.77^c^		0.438^c^
Premenopausal	297	126		90		36	
Postmenopausal	82	36		23		13	
Ultrasound score^a^ n, (%)			N/A^c^		N/A^c^		N/A^c^
0	159(42)	12 (7.4)		9		3	
1	187(49.3)	81 (50)		53		28	
2–5	33 (8.7)	69(42.6)		51		18	
CA 125 (U/mL) Mean ± SD	34.77 ± 6.16	192.15 ± 98.13	N/A^d^	235.63 ± 322.28	N/A^d^	91.8 ± 202.61	N/A^d^
Tumor size (cm)			N/A^c^		0.505^c^		N/A^c^
<7	198	58		55		3	
≥7	181	104		58		46	

^a^Ultrasound scores were recorded as one point for each of the following characteristics: multilocularity, solid areas, bilaterality, ascites and intra-abdominal metastases

^b^Student’s t-test

^c^Chi square test

^d^Mann–Whitney U test

### RMI calculation

According to RMI score algorithms (Table 1), we calculated RMI I to RMI IV for each patient by their relevant clinical data respectively. Those data were shown in Additional file 1: Table S1.

### ROC curves

**Fig. 2:**
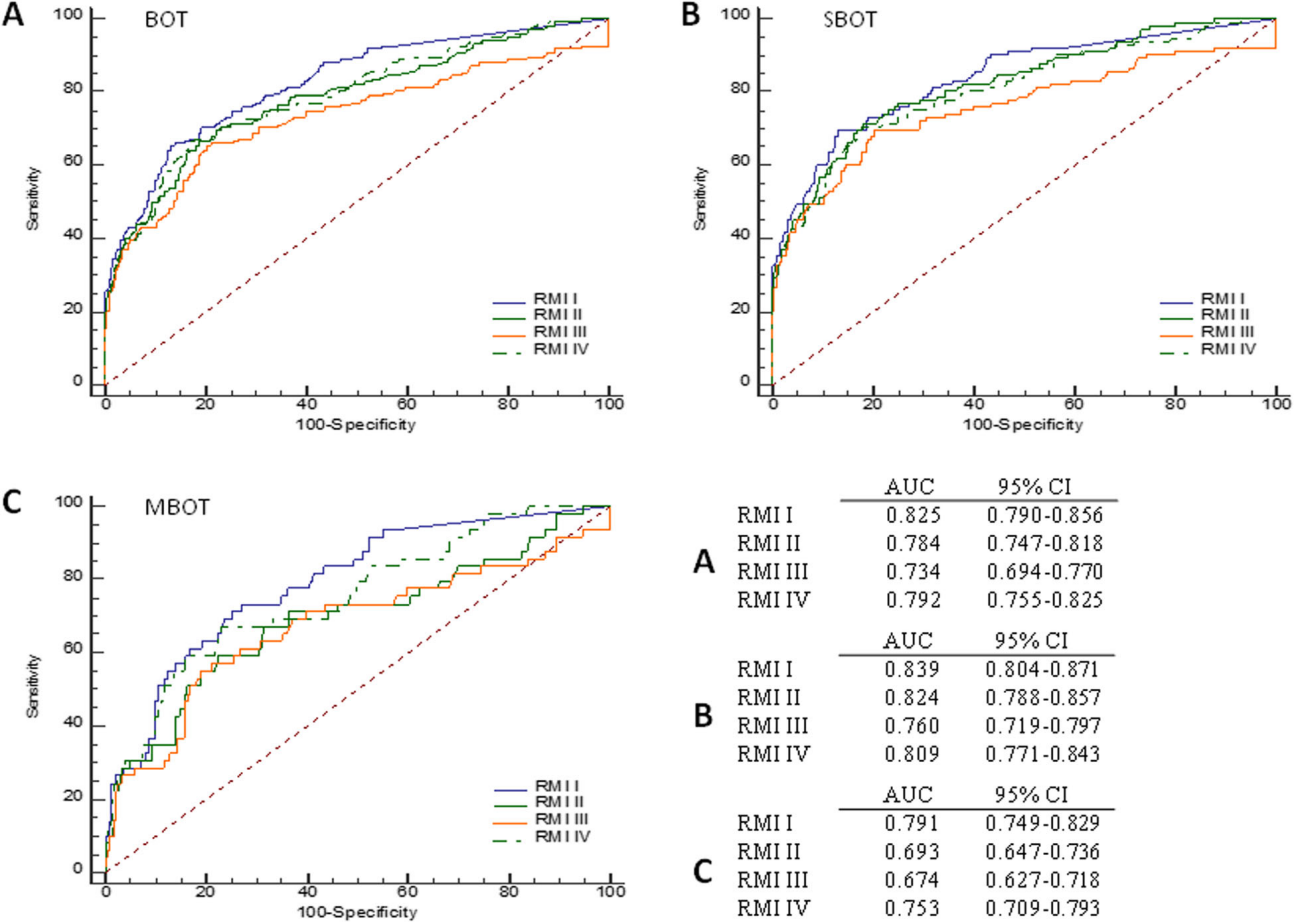
ROC curves for the detection of BOTs for RMIs in the BOTs(**a**), SBOT(**b**) and MBOT(**c**) group. Total area under the curve (AUC) values with corresponding 95% confidence intervals are listed below the curves

The ROC curves of four RMIs were shown in Fig. 2. For BOTs group, RMI I was associated with the highest AUC (0.825, 95% CI: 0.790–0.856) among all the four RMIs. Similar results were found in SBOT (0.839, 95% CI: 0.804–0.871) and MBOT (0.791, 95% CI: 0.749–0.829) subgroup. Pairwise comparison of ROC curves indicated that the AUC of RMI I was significantly larger than the AUCs of RMI II, RMI III and RMI IV (*p* = 0.0209, *p* < 0.0001 and *p* = 0.0496) in BOTs group and in MBOT subgroup (*p* < 0.0001, *p* < 0.0001 and *p* = 0.0336) (Table 4). For SBOT subgroup, the AUC between RMI I and RMI III showed significantly difference (*p* < 0.0001).

**Table 4:** Differences in the AUC of the ROC curves for the diagnosis of BOTs with the corresponding 95% confidence intervals (95% CI) and *p*-values. Pairwise ROC curve comparisons were calculated for the BOTs, SBOT and MBOT group. The method described by DeLong et al. was used to calculate the difference between two AUCs [21]

BOTs			SBOT			MBOT		
Difference	95% CI	*P*	Difference	95% CI	*P*	Difference	95% CI	*P*
I Vs II								
0.0403	0.00609–0.0745	0.0209	0.0152	−0.0272-0.0577	0.4818	0.0981	0.0572–0.139	<0.0001
I Vs III								
0.0911	0.0686–0.114	<0.0001	0.0797	0.0569–0.102	<0.0001	0.117	0.0788–0.156	<0.0001
I Vs IV								
0.033	−0.00103-0.0671	0.0496	0.0307	−0.0132-0.0746	0.1708	0.0384	0.00299–0.0739	0.0336

### Performance indices

The calculated sensitivities and specificities at the cutoff values of 60 for RMI I, II, III and 100 for RMI IV was shown in Table 5. RMI I had the highest specificity among the BOTs group (87.6, 95% CI: 83.9–90.7%), SBOT (87.6, 95% CI: 83.9–90.7%) and MBOT subgroup (87.6, 95% CI: 83.9–90.7%). RMI II scored the highest overall in terms of sensitivity among the BOTs group (69.75, 95% CI: 62.1–76.7%), SBOT (74.34, 95% CI: 65.3–82.1%) and MBOT (59.18, 95% CI: 44.2–73.0%) subgroup. In Table 6, we compared the sensitivity of RMI I, RMI III, RMI IV with RMI II in BOTs group, SBOT and MBOT subgroup. The RMI II demonstrated superior performance compared with RMI I and RMI III in BOTs (*p* = 0.002 and *p* = 0.008) and SBOT subgroup (*p* = 0.002 and *p* = 0.008), but not with RMI IV (*p* = 0.219 and *p* = 0.219).

**Table 5:** Cutoff, sensitivity, specificity, positive likelihood ratio (LR+), negative likelihood ratio (LR–) of RMI I, RMI II, RMI III and RMI IV in BOT, SBOT and MBOT group. The 95% confidence intervals (95% CI) are indicated between brackets

	Index	Cutoff	Sensitivity, %	Specificity, %	LR+	LR-
BOT	RMI I	60	63.58(55.7–71.0)	87.6(83.9–90.7)	5.13(3.8–6.9)	0.42(0.3–0.5)
	RMI II	59.7	69.75(62.1–76.7)	76.78(72.2–80.9)	3(2.4–3.7)	0.39(0.3–0.5)
	RMI III	60	63.58(55.7–71.0)	80.74(76.4–84.6)	3.3(2.6–4.2)	0.45(0.4–0.6)
	RMI IV	92	67.28(59.5–74.4)	78.1(73.6–82.2)	3.07(2.5–3.8)	0.42(0.3–0.5)
SBOT	RMI I	60	67.26(57.8–75.8)	87.6(83.9–90.7)	5.42(4.0–7.3)	0.37(0.3–0.5)
	RMI II	59.7	74.34(65.3–82.1)	76.78(72.2–80.9)	3.2(2.6–4.0)	0.33(0.2–0.5)
	RMI III	60	67.26(57.8–75.8)	80.74(76.4–84.6)	3.49(2.7–4.5)	0.41(0.3–0.5)
	RMI IV	91.8	70.8(61.5–79.0)	78.1(73.6–82.2)	3.23(2.6–4.0)	0.37(0.3–0.5)
MBOT	RMI I	60	55.1(40.2–69.3)	87.6(83.9–90.7)	4.44(3.1–6.4)	0.51(0.4–0.7)
	RMI II	61	59.18(44.2–73.0)	77.57(73.0–81.7)	2.64(2.0–3.6)	0.53(0.4–0.7)
	RMI III	61	55.1(40.2–69.3)	81(76.7–84.8)	2.9(2.1–4.0)	0.55(0.4–0.8)
	RMI IV	92	59.18(44.2–73.0)	78.1(73.6–82.2)	2.7(2.0–3.6)	0.52(0.4–0.7)

**Table 6:** The sensitivity of RMI I, RMI III, and RMI IV compared with RMI II

	RMI II	RMI I		RMI III		RMI IV	
	Sensitivity	Sensitivity	*P*	Sensitivity	*P*	Sensitivity	*P*
BOT	69.75(62.1–76.7)	63.58(55.7–71.0)	0.002	63.58(55.7–71.0)	0.002	67.28(59.5–74.4)	0.219
SBOT	74.34(65.3–82.1)	67.26(57.8–75.8)	0.008	67.26(57.8–75.8)	0.008	70.8(61.5–79.0)	0.219
MBOT	59.18(44.2–73.0)	55.1(40.2–69.3)	0.5	55.1(40.2–69.3)	0.5	59.18(44.2–73.0)	1

## Discussion

**Fig. 3:**
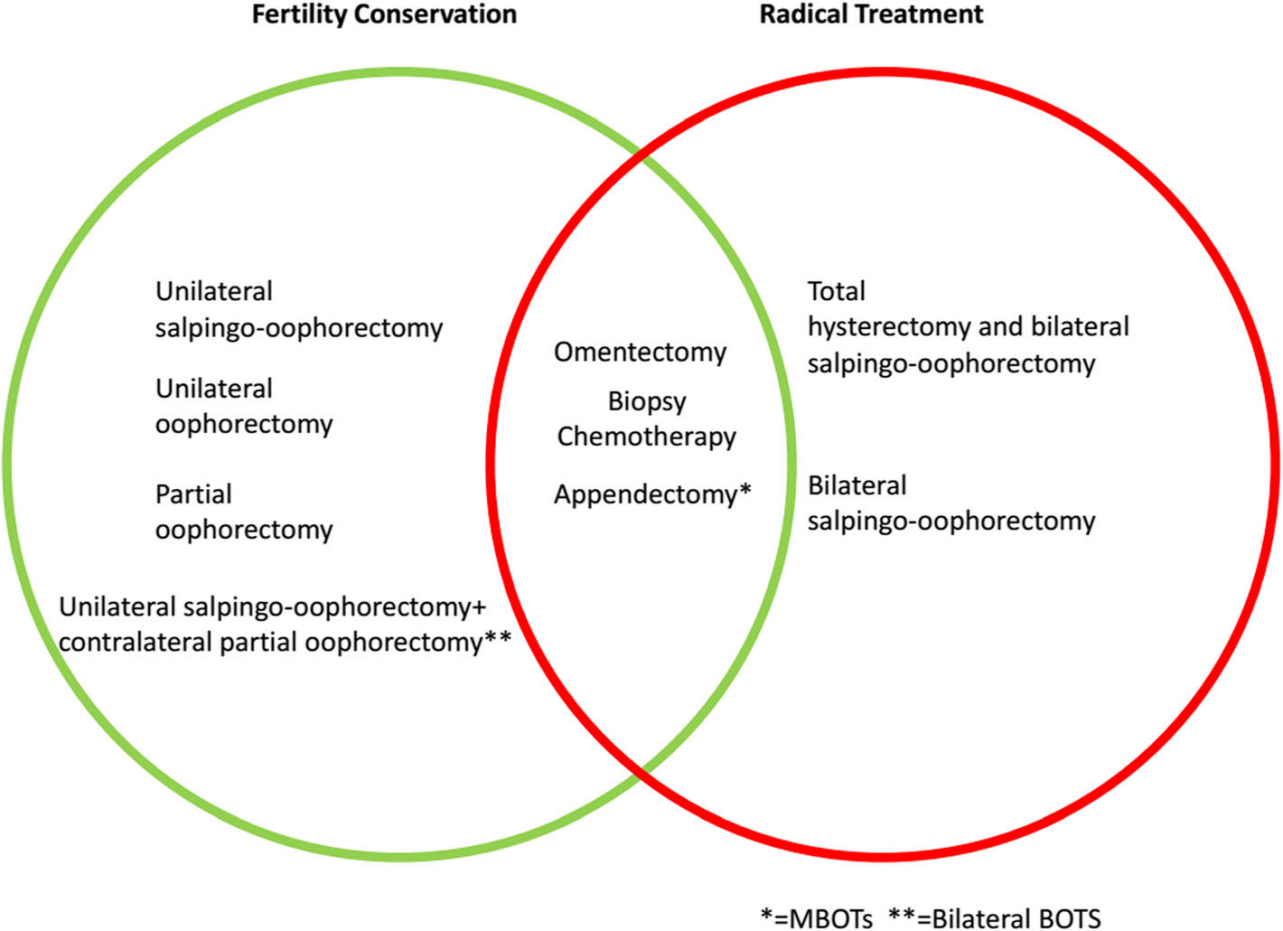
Recommended surgery method for ovarian borderline tumor patients with/without the willingness to preserve fertility

In the 1990s, Jacobs et al. originally developed the RMI, which is known as RMI I [22]. Modifying RMI, Tingulstad et al. developed RMI II and III, with the alternation of the ratio of ultrasound score and postmenopausal status score [23, 24]. Recently RMI IV was created by Yamamoto et al. by adding the parameter of the tumor size [25]. Over the past few years, the performance of RMI to distinguish benign from malignant adnexal masses has been well studied. However, how to discriminate borderline ovarian tumors from benign ovarian tumors has been in great difficulty over years, as BOTs present less typical tumor features [26, 27]. In fact, the preoperative discrimination is quite important for BOTs, as the recommended surgery methods are different (Fig. 3). Our study has revealed the effectiveness of using RMIs to predict tumor nature, which could help both surgeon and pathologist making pre and in operation decision for proper treatment to benefit patients, especially who wish to preserve their reproductive capacity before the operation.

In previous studies, BOTs are not evaluated as a separate group and usually included in malignant groups, but their clinical features are more easily to be confused with benign ones. Although the clinical outcome is good, there are still many advanced cases. For the reason above, we applied these RMIs only between BOTs and benign lesions to assess RMIs performance in the differential diagnosis. Our results show that RMI I conducted the best performance in BOTs group, SBOT, and MBOT subgroups. The AUCs of the RMI I were 0.825, 0.839 and 0.791 respectively. It suggests that RMI I was the best method to differentiate BOTs from benign ovarian tumors. Moreover, we found that the AUCs of four RMIs in BOTs and SBOT group were both more than 0.7, it implies that RMIs are possible to identify SBOT before the operation. However, in MBOT group, the AUCs of four RMIs were smaller, especially for the RMI II and RMI III, which were both less than 0.7. Gotlieb et al. showed elevated CA125 concentrations in 75% of SBOT and only 30% of MBOT [10]. This may partly account for the poor performance of RMIs in discriminating MBOTs and benign ovarian masses. Regards of the sensitivity, we found RMI II was the highest for BOTs group, SBOT, and MBOT subgroups. However, there is a risk of use RMI II, as it provides more weighting to the ultrasound findings when compared to RMI I, RMI III and RMI IV. This also explains the improved sensitivity in RMI II. In MBOT subgroup, the sensitivity of RMI II and RMI IV were similar and better than other groups. The most significant factor is that RMI IV included a new parameter about the tumor size. From the previous study, we know that MBOTs demonstrate a significantly larger tumor size than SBOTs [28]. Taken all together, the specificity of RMI I was the highest in all the three groups. The cutoff of the previous studies which investigated the difference between benign and malignant ovarian tumors is 200 for RIM I, RMI II and RMI III [22–24]. The suggestive cutoff for RMI IV is 450 [25]. However, in our study, all the values of the cutoff for the four RMIs are relatively lower. The main reason is that the ultrasound score, CA125, the percent of postmenopausal status and tumor size of BOTs are lower than those of malignant ovarian tumor. The cutoff of RMI I, II and III is about 60, and 100 for RMI IV. As RMI I may take the best performance of distinguishing BOTs from benign tumors, considering its application in malignancy, we may use < 60, 60–200, > 200 as warning lines for clinicians.

Since elevated levels of CA19–9 have been reported in BOT, especially in mucinous histological types [10, 27], measurement of CA19–9 has been proposed to be of some clinical value in combination with CA125 as a marker for serological monitoring of BOT [29]. Accordingly, in some institutions, CA19–9 has been incorporated as a tumor marker for evaluation of patients with adnexal masses. However, none of the national guidelines, including those of the American College of Obstetricians and Gynecologists (ACOG), the Society of Gynecologic Oncologists (SGO) and National Institute for Health and Clinical Excellence (NICE), have included CA19–9 measurement as an adjunct in the triage of patients with adnexal masses [30, 31]. Alanbay I et al. have conducted a meaningful study. They modified the RMI IV formulation, replacing CA125 with CA19–9. Then they compared RMI IV (CA125), RMI IV (CA19–9), serum CA125 and CA19–9 level, ultrasound score, and menopausal status between BOTs and benign adnexal masses. They found the sensitivity of CA 19–9 (40%) lower than CA 125(54%). RMI IV (CA125) was found to be the best predictive method for differentiation of BOTs from benign adnexal masses. Replacing CA125 with CA19–9 didn’t affect RMI IV sensitivity and specificity for discrimination between BOTs and benign adnexal masses [17]. It indicates that CA125 is more important in discrimination between BOTs and benign adnexal masses, or it is appropriate for RMI than CA19–9. Moreover, the level of CA19–9 was shown to be high in several benign ovarian findings, especially mature cystic teratomas [32], and even in nongynecological conditions such as rheumatoid arthritis [33]. Several studies found increased CA19–9 levels in 37.4–39.6% of mature cystic teratomas cases [34, 35]. It may affect the accuracy of discrimination between BOT and benign ovarian tumors. From what has been discussed above, we selected CA125 instead of CA19–9 as a one of the parameters of RMI.

The evaluation of strategies for the BOTs has not been considered by histologic subtype in previous studies, or even with results that it is impossible to distinguish benign tumor from BOTs. Our study has its own limitations that we only classify BOTs into SBOT and MBOT subgroups and more in-depth clinical studies with the large patient number should be added for validation. Also, the ultrasound findings are greatly influenced by the sonographer. However, we hope that our study would be able to solve certain preoperation question raised in borderline ovarian tumors, especially as a potent reminder for the clinicians. .

## Supplementary information

**Additional file 1: Table S1.** Group: 1 represents benign ovarian tumor, 2 represents BOT. M: 0 represents premenopausal status, 1 represents postmenopausal status. U represents ultrasound score.

## Data Availability

All data were included in this article.

## Abbreviations

AUC: Area under the receiver operator characteristic curves
BOTs: Borderline ovarian tumors
MBOTs: Mucinous borderline ovarian tumors
RMI: Risk of malignancy index
SBOTs: Serous borderline ovarian tumors
