# Supplementary material for: Diagnostic extended usefulness of RMI: comparison of four risk of malignancy index in preoperative differentiation of borderline ovarian tumors and benign ovarian tumors
Source: J Ovarian Res. 2019 Sep 16;12:87. doi: 10.1186/s13048-019-0568-3 (PMC6747741; doi:10.1186/s13048-019-0568-3)
Supplement: Supplementary file 1 — Additional file 1: Table S1. Group: 1 represents benign ovarian tumor, 2 represents BOT. M: 0 represents premenopausal status, 1 represents postmenopausal status. U represents ultrasound score. [file 13048_2019_568_MOESM1_ESM.docx]

Additional file 1: Table S1. Group: 1 represents benign ovarian tumor, 2 represents BOT. M: 0 represents premenopausal status, 1 represents postmenopausal status. U represents ultrasound score

| Group | RMI I | RMI II | RMI III | RMI IV | CA125(u/ml) | M | U | Tumor size(cm) |
| --- | --- | --- | --- | --- | --- | --- | --- | --- |
| 1 | 15.8 | 15.8 | 15.8 | 31.6 | 15.8 | 0 | 1 | 15.5 |
| 1 | 33.9 | 45.2 | 33.9 | 90.4 | 11.3 | 1 | 1 | 12.2 |
| 1 | 38.3 | 38.3 | 38.3 | 76.6 | 38.3 | 0 | 1 | 7.5 |
| 1 | 625.8 | 834.4 | 625.8 | 1668.8 | 208.6 | 1 | 1 | 10.1 |
| 1 | 123.3 | 164.4 | 123.3 | 164.4 | 41.1 | 1 | 1 | 4.4 |
| 1 | 65.64 | 87.52 | 65.64 | 175.04 | 21.88 | 0 | 2 | 18.7 |
| 1 | 11.5 | 11.5 | 11.5 | 23 | 11.5 | 0 | 1 | 9.6 |
| 1 | 285.3 | 507.2 | 285.3 | 1014.4 | 31.7 | 1 | 2 | 14.1 |
| 1 | 75.3 | 100.4 | 75.3 | 200.8 | 25.1 | 0 | 2 | 16 |
| 1 | 184.9 | 184.9 | 184.9 | 184.9 | 184.9 | 0 | 1 | 4.3 |
| 1 | 474 | 632 | 474 | 632 | 158 | 0 | 2 | 5.1 |
| 1 | 384.6 | 512.8 | 384.6 | 1025.6 | 128.2 | 0 | 2 | 13.8 |
| 1 | 26.7 | 26.7 | 26.7 | 53.4 | 26.7 | 0 | 1 | 8.4 |
| 1 | 0 | 12.4 | 0 | 24.8 | 12.4 | 0 | 0 | 7.9 |
| 1 | 61.3 | 61.3 | 61.3 | 122.6 | 61.3 | 0 | 1 | 16.5 |
| 1 | 25.7 | 25.7 | 25.7 | 25.7 | 25.7 | 0 | 1 | 4.6 |
| 1 | 70.2 | 93.6 | 70.2 | 187.2 | 23.4 | 1 | 1 | 30.3 |
| 1 | 165.9 | 221.2 | 165.9 | 221.2 | 55.3 | 0 | 3 | 6.9 |
| 1 | 21.4 | 21.4 | 21.4 | 42.8 | 21.4 | 0 | 1 | 9.8 |
| 1 | 54 | 72 | 54 | 144 | 18 | 1 | 1 | 16.2 |
| 1 | 82.9 | 82.9 | 82.9 | 165.8 | 82.9 | 0 | 1 | 16 |
| 1 | 10.6 | 10.6 | 10.6 | 21.2 | 10.6 | 0 | 1 | 15.5 |
| 1 | 13.5 | 13.5 | 13.5 | 13.5 | 13.5 | 0 | 1 | 4.9 |
| 1 | 1198.5 | 1598 | 1198.5 | 1598 | 399.5 | 0 | 2 | 5 |
| 1 | 29.2 | 29.2 | 29.2 | 29.2 | 29.2 | 0 | 1 | 6.1 |
| 1 | 3000 | 4000 | 3000 | 8000 | 1000 | 0 | 2 | 15.4 |
| 1 | 79.8 | 79.8 | 79.8 | 159.6 | 79.8 | 0 | 1 | 8.8 |
| 1 | 57.6 | 76.8 | 57.6 | 76.8 | 19.2 | 0 | 2 | 5 |
| 1 | 863.4 | 1151.2 | 863.4 | 1151.2 | 287.8 | 1 | 1 | 5.6 |
| 1 | 0 | 116.7 | 0 | 116.7 | 116.7 | 0 | 0 | 5 |
| 1 | 35.4 | 47.2 | 35.4 | 47.2 | 11.8 | 1 | 1 | 3.8 |
| 1 | 301.5 | 402 | 301.5 | 804 | 100.5 | 0 | 3 | 10 |
| 1 | 23.2 | 23.2 | 23.2 | 23.2 | 23.2 | 0 | 1 | 3.6 |
| 1 | 696.3 | 928.4 | 696.3 | 1856.8 | 232.1 | 0 | 2 | 11.5 |
| 1 | 564.9 | 753.2 | 564.9 | 1506.4 | 188.3 | 1 | 1 | 25 |
| 1 | 57.9 | 77.2 | 57.9 | 154.4 | 19.3 | 1 | 1 | 7.8 |
| 1 | 1481.7 | 1975.6 | 1481.7 | 3951.2 | 493.9 | 0 | 3 | 13.3 |
| 1 | 68.7 | 91.6 | 68.7 | 183.2 | 22.9 | 0 | 2 | 9.8 |
| 1 | 1544.7 | 2059.6 | 1544.7 | 4119.2 | 514.9 | 0 | 2 | 8.1 |
| 1 | 3000 | 4000 | 3000 | 8000 | 1000 | 0 | 2 | 7.9 |
| 1 | 2340 | 3120 | 2340 | 6240 | 780 | 0 | 2 | 10 |
| 1 | 215.7 | 287.6 | 215.7 | 575.2 | 71.9 | 0 | 2 | 15.8 |
| 1 | 0 | 1838.8 | 0 | 3677.6 | 459.7 | 1 | 0 | 11 |
| 1 | 285.7 | 285.7 | 285.7 | 571.4 | 285.7 | 0 | 1 | 33.3 |
| 1 | 27.7 | 27.7 | 27.7 | 55.4 | 27.7 | 0 | 1 | 7.7 |
| 1 | 9000 | 16000 | 9000 | 16000 | 1000 | 1 | 4 | 2.2 |
| 1 | 1000 | 1000 | 1000 | 1000 | 1000 | 0 | 1 | 6.6 |
| 1 | 97.2 | 129.6 | 97.2 | 259.2 | 32.4 | 0 | 2 | 7.1 |
| 1 | 21.9 | 21.9 | 21.9 | 43.8 | 21.9 | 0 | 1 | 8.1 |
| 1 | 10.6 | 10.6 | 10.6 | 21.2 | 10.6 | 0 | 1 | 7.8 |
| 1 | 539.1 | 958.4 | 539.1 | 958.4 | 59.9 | 1 | 2 | 5.7 |
| 1 | 150.3 | 267.2 | 150.3 | 534.4 | 16.7 | 1 | 2 | 7.5 |
| 1 | 753.3 | 1004.4 | 753.3 | 2008.8 | 251.1 | 1 | 1 | 9 |
| 1 | 0 | 3147.2 | 0 | 6294.4 | 786.8 | 1 | 0 | 18.2 |
| 1 | 41.1 | 41.1 | 41.1 | 41.1 | 41.1 | 0 | 1 | 6.6 |
| 1 | 3000 | 4000 | 3000 | 8000 | 1000 | 0 | 2 | 12 |
| 1 | 16.6 | 16.6 | 16.6 | 33.2 | 16.6 | 0 | 1 | 8.1 |
| 1 | 59.7 | 59.7 | 59.7 | 59.7 | 59.7 | 0 | 1 | 5.9 |
| 1 | 1470.9 | 1961.2 | 1470.9 | 3922.4 | 490.3 | 0 | 3 | 23.6 |
| 1 | 91.8 | 91.8 | 91.8 | 91.8 | 91.8 | 0 | 1 | 4.1 |
| 1 | 62.2 | 62.2 | 62.2 | 124.4 | 62.2 | 0 | 1 | 7.6 |
| 1 | 1252.8 | 1670.4 | 1252.8 | 3340.8 | 417.6 | 0 | 2 | 13.3 |
| 1 | 34.5 | 46 | 34.5 | 92 | 11.5 | 1 | 1 | 12.6 |
| 1 | 1406.7 | 1875.6 | 1406.7 | 1875.6 | 468.9 | 0 | 4 | 6.8 |
| 1 | 3000 | 4000 | 3000 | 8000 | 1000 | 0 | 3 | 10.5 |
| 1 | 18.8 | 18.8 | 18.8 | 37.6 | 18.8 | 0 | 1 | 9 |
| 1 | 70.7 | 70.7 | 70.7 | 141.4 | 70.7 | 0 | 1 | 11.5 |
| 1 | 16.5 | 16.5 | 16.5 | 16.5 | 16.5 | 0 | 1 | 5.7 |
| 1 | 61.5 | 82 | 61.5 | 82 | 20.5 | 0 | 2 | 4.5 |
| 1 | 87.3 | 116.4 | 87.3 | 232.8 | 29.1 | 0 | 2 | 10.7 |
| 1 | 76.5 | 102 | 76.5 | 204 | 25.5 | 0 | 2 | 8.4 |
| 1 | 40.9 | 40.9 | 40.9 | 81.8 | 40.9 | 0 | 1 | 7.5 |
| 1 | 0 | 165.2 | 0 | 330.4 | 41.3 | 1 | 0 | 10.2 |
| 1 | 46.8 | 62.4 | 46.8 | 124.8 | 15.6 | 0 | 2 | 10.3 |
| 1 | 83.1 | 110.8 | 83.1 | 110.8 | 27.7 | 0 | 2 | 3 |
| 1 | 482.1 | 642.8 | 482.1 | 1285.6 | 160.7 | 0 | 2 | 16.9 |
| 1 | 3063.6 | 5446.4 | 3063.6 | 5446.4 | 340.4 | 1 | 2 | 5.3 |
| 1 | 60.9 | 81.2 | 60.9 | 162.4 | 20.3 | 1 | 1 | 8.9 |
| 1 | 8.6 | 8.6 | 8.6 | 17.2 | 8.6 | 0 | 1 | 15.2 |
| 1 | 452.7 | 804.8 | 452.7 | 804.8 | 50.3 | 1 | 2 | 5.4 |
| 1 | 317.7 | 423.6 | 317.7 | 423.6 | 105.9 | 1 | 1 | 5.9 |
| 1 | 0 | 12.9 | 0 | 25.8 | 12.9 | 0 | 0 | 9.3 |
| 1 | 19.6 | 19.6 | 19.6 | 19.6 | 19.6 | 0 | 1 | 6.18 |
| 1 | 19.06 | 19.06 | 19.06 | 38.12 | 19.06 | 0 | 1 | 7.2 |
| 1 | 38.7 | 38.7 | 38.7 | 38.7 | 38.7 | 0 | 1 | 3.4 |
| 1 | 2902.8 | 3870.4 | 2902.8 | 7740.8 | 967.6 | 0 | 3 | 18.5 |
| 1 | 877.2 | 1169.6 | 877.2 | 2339.2 | 292.4 | 1 | 1 | 13.8 |
| 1 | 0 | 93.5 | 0 | 187 | 93.5 | 0 | 0 | 9.9 |
| 1 | 164.1 | 218.8 | 164.1 | 437.6 | 54.7 | 0 | 2 | 8.3 |
| 1 | 554.4 | 985.6 | 554.4 | 985.6 | 61.6 | 1 | 2 | 6.5 |
| 1 | 3000 | 4000 | 3000 | 8000 | 1000 | 1 | 1 | 20 |
| 1 | 24.6 | 32.8 | 24.6 | 32.8 | 8.2 | 1 | 1 | 5.3 |
| 1 | 24.8 | 24.8 | 24.8 | 24.8 | 24.8 | 0 | 1 | 4 |
| 1 | 18 | 18 | 18 | 36 | 18 | 0 | 1 | 16.6 |
| 1 | 108.6 | 144.8 | 108.6 | 144.8 | 36.2 | 0 | 2 | 6.8 |
| 1 | 137.5 | 137.5 | 137.5 | 137.5 | 137.5 | 0 | 1 | 4.8 |
| 1 | 3000 | 4000 | 3000 | 8000 | 1000 | 0 | 3 | 14.7 |
| 1 | 97.8 | 130.4 | 97.8 | 260.8 | 32.6 | 0 | 2 | 24 |
| 1 | 2134.8 | 2846.4 | 2134.8 | 5692.8 | 711.6 | 0 | 3 | 13 |
| 1 | 202.5 | 270 | 202.5 | 540 | 67.5 | 0 | 4 | 10.8 |
| 1 | 73.8 | 131.2 | 73.8 | 262.4 | 8.2 | 1 | 2 | 13.4 |
| 1 | 76.2 | 101.6 | 76.2 | 203.2 | 25.4 | 0 | 2 | 24 |
| 1 | 29.1 | 38.8 | 29.1 | 38.8 | 9.7 | 0 | 2 | 5 |
| 1 | 129.6 | 172.8 | 129.6 | 172.8 | 43.2 | 0 | 2 | 5.2 |
| 1 | 15.8 | 15.8 | 15.8 | 15.8 | 15.8 | 0 | 1 | 5 |
| 1 | 440.4 | 440.4 | 440.4 | 440.4 | 440.4 | 0 | 1 | 3.5 |
| 1 | 0 | 20.1 | 0 | 40.2 | 20.1 | 0 | 0 | 8.2 |
| 1 | 41.1 | 54.8 | 41.1 | 109.6 | 13.7 | 0 | 2 | 9.6 |
| 1 | 94.2 | 94.2 | 94.2 | 188.4 | 94.2 | 0 | 1 | 13.2 |
| 1 | 102.9 | 137.2 | 102.9 | 274.4 | 34.3 | 0 | 3 | 8 |
| 1 | 62.7 | 83.6 | 62.7 | 167.2 | 20.9 | 1 | 1 | 13.6 |
| 1 | 75.3 | 100.4 | 75.3 | 200.8 | 25.1 | 1 | 1 | 15.3 |
| 1 | 162 | 288 | 162 | 288 | 18 | 1 | 2 | 4.5 |
| 1 | 319.2 | 425.6 | 319.2 | 851.2 | 106.4 | 0 | 2 | 21 |
| 1 | 0 | 55.5 | 0 | 55.5 | 55.5 | 0 | 0 | 6.6 |
| 1 | 6318 | 11232 | 6318 | 22464 | 702 | 1 | 3 | 14.7 |
| 1 | 143.5 | 143.5 | 143.5 | 287 | 143.5 | 0 | 1 | 7.4 |
| 1 | 252.6 | 336.8 | 252.6 | 673.6 | 84.2 | 0 | 2 | 28.4 |
| 1 | 17.2 | 17.2 | 17.2 | 17.2 | 17.2 | 0 | 1 | 5.3 |
| 1 | 31.6 | 31.6 | 31.6 | 63.2 | 31.6 | 0 | 1 | 22.8 |
| 1 | 182.1 | 242.8 | 182.1 | 485.6 | 60.7 | 1 | 1 | 20 |
| 1 | 1146 | 1146 | 1146 | 2292 | 1146 | 0 | 1 | 8.5 |
| 1 | 286.2 | 381.6 | 286.2 | 763.2 | 95.4 | 0 | 2 | 14.4 |
| 1 | 104.4 | 185.6 | 104.4 | 371.2 | 11.6 | 1 | 2 | 14.4 |
| 1 | 62.1 | 82.8 | 62.1 | 165.6 | 20.7 | 0 | 3 | 7 |
| 1 | 21.1 | 21.1 | 21.1 | 42.2 | 21.1 | 0 | 1 | 32.5 |
| 1 | 229.3 | 229.3 | 229.3 | 458.6 | 229.3 | 0 | 1 | 16 |
| 1 | 66.2 | 66.2 | 66.2 | 66.2 | 66.2 | 0 | 1 | 6.8 |
| 1 | 112.8 | 112.8 | 112.8 | 112.8 | 112.8 | 0 | 1 | 6.6 |
| 1 | 179.7 | 239.6 | 179.7 | 479.2 | 59.9 | 1 | 1 | 8.8 |
| 1 | 33.4 | 33.4 | 33.4 | 33.4 | 33.4 | 0 | 1 | 5 |
| 1 | 0 | 132 | 0 | 132 | 33 | 1 | 0 | 2.7 |
| 1 | 643.8 | 858.4 | 643.8 | 1716.8 | 214.6 | 0 | 2 | 9 |
| 1 | 143.9 | 143.9 | 143.9 | 143.9 | 143.9 | 0 | 1 | 6.2 |
| 1 | 832.6 | 832.6 | 832.6 | 832.6 | 832.6 | 0 | 1 | 4.5 |
| 1 | 16.2 | 16.2 | 16.2 | 16.2 | 16.2 | 0 | 1 | 3.1 |
| 1 | 2487.9 | 3317.2 | 2487.9 | 6634.4 | 829.3 | 0 | 3 | 12.1 |
| 1 | 110.7 | 147.6 | 110.7 | 295.2 | 36.9 | 0 | 2 | 7.7 |
| 1 | 33.6 | 44.8 | 33.6 | 89.6 | 11.2 | 0 | 2 | 20 |
| 1 | 106 | 106 | 106 | 212 | 106 | 0 | 1 | 12.3 |
| 1 | 542.7 | 964.8 | 542.7 | 1929.6 | 60.3 | 1 | 2 | 25 |
| 1 | 44.3 | 44.3 | 44.3 | 88.6 | 44.3 | 0 | 1 | 15.1 |
| 1 | 726.6 | 968.8 | 726.6 | 1937.6 | 242.2 | 0 | 2 | 20.5 |
| 1 | 89.4 | 119.2 | 89.4 | 119.2 | 29.8 | 0 | 2 | 6.9 |
| 1 | 165 | 220 | 165 | 440 | 55 | 0 | 3 | 8.9 |
| 1 | 0 | 33.6 | 0 | 33.6 | 33.6 | 0 | 0 | 4.3 |
| 1 | 0 | 37.2 | 0 | 37.2 | 37.2 | 0 | 0 | 6.5 |
| 1 | 3000 | 4000 | 3000 | 8000 | 1000 | 0 | 3 | 16.4 |
| 1 | 273.9 | 365.2 | 273.9 | 730.4 | 91.3 | 0 | 2 | 9 |
| 1 | 1320.6 | 1760.8 | 1320.6 | 1760.8 | 440.2 | 0 | 2 | 4.9 |
| 1 | 479.3 | 479.3 | 479.3 | 479.3 | 479.3 | 0 | 1 | 6.4 |
| 1 | 3000 | 4000 | 3000 | 8000 | 1000 | 0 | 2 | 12.6 |
| 1 | 82 | 82 | 82 | 164 | 82 | 0 | 1 | 30 |
| 1 | 31.8 | 42.4 | 31.8 | 42.4 | 10.6 | 0 | 2 | 5.7 |
| 1 | 12.5 | 12.5 | 12.5 | 25 | 12.5 | 0 | 1 | 11.2 |
| 1 | 357.7 | 357.7 | 357.7 | 357.7 | 357.7 | 0 | 1 | 6.2 |
| 1 | 11.3 | 11.3 | 11.3 | 11.3 | 11.3 | 0 | 1 | 4.7 |
| 1 | 1000 | 1000 | 1000 | 2000 | 1000 | 0 | 1 | 12 |
| 1 | 179.7 | 179.7 | 179.7 | 179.7 | 179.7 | 0 | 1 | 2.1 |
| 1 | 197.7 | 263.6 | 197.7 | 263.6 | 65.9 | 0 | 2 | 4.9 |
| 1 | 18.2 | 18.2 | 18.2 | 36.4 | 18.2 | 0 | 1 | 12.2 |
| 1 | 66.5 | 66.5 | 66.5 | 66.5 | 66.5 | 0 | 1 | 6.1 |
| 2 | 0 | 9.8 | 9.8 | 19.6 | 9.8 | 0 | 0 | 8.3 |
| 2 | 22.5 | 30 | 22.5 | 60 | 7.5 | 1 | 1 | 13 |
| 2 | 0 | 16.1 | 16.1 | 16.1 | 16.1 | 0 | 0 | 6.5 |
| 2 | 33.3 | 44.4 | 33.3 | 88.8 | 11.1 | 1 | 1 | 11.7 |
| 2 | 0 | 12.8 | 12.8 | 12.8 | 12.8 | 0 | 0 | 6.5 |
| 2 | 6.7 | 6.7 | 6.7 | 6.7 | 6.7 | 0 | 1 | 6.5 |
| 2 | 25.5 | 34 | 25.5 | 68 | 8.5 | 1 | 1 | 14.8 |
| 2 | 0 | 11.1 | 11.1 | 22.2 | 11.1 | 0 | 0 | 13.1 |
| 2 | 16 | 16 | 16 | 32 | 16 | 0 | 1 | 9.3 |
| 2 | 13 | 13 | 13 | 13 | 13 | 0 | 1 | 6.6 |
| 2 | 23.6 | 23.6 | 23.6 | 47.2 | 23.6 | 0 | 1 | 10.6 |
| 2 | 0 | 14.8 | 14.8 | 29.6 | 14.8 | 0 | 0 | 8.5 |
| 2 | 14.3 | 14.3 | 14.3 | 14.3 | 14.3 | 0 | 1 | 4.8 |
| 2 | 0 | 17.7 | 17.7 | 17.7 | 17.7 | 0 | 0 | 5.6 |
| 2 | 18.9 | 25.2 | 18.9 | 50.4 | 6.3 | 1 | 1 | 7.1 |
| 2 | 19.8 | 26.4 | 19.8 | 26.4 | 6.6 | 1 | 1 | 6.7 |
| 2 | 0 | 121 | 121 | 121 | 121 | 0 | 0 | 6.7 |
| 2 | 102.6 | 136.8 | 102.6 | 273.6 | 34.2 | 1 | 1 | 18 |
| 2 | 43.2 | 76.8 | 43.2 | 153.6 | 4.8 | 1 | 2 | 7.6 |
| 2 | 21 | 28 | 21 | 56 | 7 | 1 | 1 | 18.6 |
| 2 | 19.3 | 19.3 | 19.3 | 38.6 | 19.3 | 0 | 1 | 7.2 |
| 2 | 18 | 18 | 18 | 36 | 18 | 0 | 1 | 12.9 |
| 2 | 0 | 31.5 | 31.5 | 31.5 | 31.5 | 0 | 0 | 6.6 |
| 2 | 0 | 11.1 | 11.1 | 22.2 | 11.1 | 0 | 0 | 14.1 |
| 2 | 0 | 15.7 | 15.7 | 31.4 | 15.7 | 0 | 0 | 10.9 |
| 2 | 0 | 35.6 | 26.7 | 35.6 | 8.9 | 1 | 0 | 6.8 |
| 2 | 0 | 12 | 12 | 24 | 12 | 0 | 0 | 9.4 |
| 2 | 0 | 53.2 | 39.9 | 53.2 | 13.3 | 1 | 0 | 6.9 |
| 2 | 20.7 | 20.7 | 20.7 | 41.4 | 20.7 | 0 | 1 | 13.1 |
| 2 | 14.1 | 14.1 | 14.1 | 14.1 | 14.1 | 0 | 1 | 5.5 |
| 2 | 0 | 21 | 21 | 42 | 21 | 0 | 0 | 7.5 |
| 2 | 0 | 19.2 | 14.4 | 19.2 | 4.8 | 1 | 0 | 5.9 |
| 2 | 0 | 34.5 | 34.5 | 69 | 34.5 | 0 | 0 | 7.4 |
| 2 | 0 | 8.6 | 8.6 | 17.2 | 8.6 | 0 | 0 | 11.3 |
| 2 | 18.7 | 18.7 | 18.7 | 37.4 | 18.7 | 0 | 1 | 11.6 |
| 2 | 27.6 | 36.8 | 27.6 | 73.6 | 9.2 | 1 | 1 | 10.9 |
| 2 | 0 | 152 | 114 | 304 | 38 | 1 | 0 | 25 |
| 2 | 21 | 21 | 21 | 21 | 21 | 0 | 1 | 6.6 |
| 2 | 37.7 | 37.7 | 37.7 | 75.4 | 37.7 | 0 | 1 | 13.3 |
| 2 | 12.5 | 12.5 | 12.5 | 12.5 | 12.5 | 0 | 1 | 5.8 |
| 2 | 0 | 22.9 | 22.9 | 22.9 | 22.9 | 0 | 0 | 6.8 |
| 2 | 0 | 16.1 | 16.1 | 16.1 | 16.1 | 0 | 0 | 5.2 |
| 2 | 48.3 | 64.4 | 48.3 | 64.4 | 16.1 | 0 | 2 | 5.6 |
| 2 | 29.7 | 39.6 | 29.7 | 79.2 | 9.9 | 0 | 2 | 7.8 |
| 2 | 0 | 29.9 | 29.9 | 29.9 | 29.9 | 0 | 0 | 6.8 |
| 2 | 9.5 | 9.5 | 9.5 | 19 | 9.5 | 0 | 1 | 8.7 |
| 2 | 23.1 | 23.1 | 23.1 | 23.1 | 23.1 | 0 | 1 | 6.8 |
| 2 | 18.6 | 24.8 | 18.6 | 24.8 | 6.2 | 1 | 1 | 5.9 |
| 2 | 17.6 | 17.6 | 17.6 | 35.2 | 17.6 | 0 | 1 | 7.9 |
| 2 | 116 | 116 | 116 | 232 | 116 | 0 | 1 | 10.2 |
| 2 | 0 | 69.6 | 52.2 | 139.2 | 17.4 | 1 | 0 | 15.2 |
| 2 | 0 | 35.2 | 26.4 | 70.4 | 8.8 | 1 | 0 | 7.5 |
| 2 | 9 | 9 | 9 | 18 | 9 | 0 | 1 | 9.4 |
| 2 | 0 | 26 | 26 | 52 | 26 | 0 | 0 | 14.6 |
| 2 | 0 | 48 | 36 | 96 | 12 | 1 | 0 | 10.5 |
| 2 | 35.7 | 47.6 | 35.7 | 95.2 | 11.9 | 1 | 1 | 16.7 |
| 2 | 0 | 40 | 30 | 80 | 10 | 1 | 0 | 7.9 |
| 2 | 0 | 42.8 | 32.1 | 42.8 | 10.7 | 1 | 0 | 5.1 |
| 2 | 0 | 45.2 | 33.9 | 90.4 | 11.3 | 1 | 0 | 7.3 |
| 2 | 30 | 40 | 30 | 80 | 10 | 1 | 1 | 9.3 |
| 2 | 36.6 | 48.8 | 36.6 | 48.8 | 12.2 | 1 | 1 | 4.5 |
| 2 | 7.6 | 7.6 | 7.6 | 7.6 | 7.6 | 0 | 1 | 6.5 |
| 2 | 13.7 | 13.7 | 13.7 | 27.4 | 13.7 | 0 | 1 | 8.2 |
| 2 | 46.2 | 61.6 | 46.2 | 61.6 | 15.4 | 1 | 1 | 3.8 |
| 2 | 0 | 15 | 15 | 30 | 15 | 0 | 0 | 36.6 |
| 2 | 0 | 28.8 | 21.6 | 57.6 | 7.2 | 1 | 0 | 9.5 |
| 2 | 0 | 44.4 | 33.3 | 88.8 | 11.1 | 1 | 0 | 14.1 |
| 2 | 116.1 | 206.4 | 116.1 | 412.8 | 12.9 | 1 | 2 | 11.2 |
| 2 | 96 | 128 | 96 | 256 | 32 | 1 | 1 | 25.5 |
| 2 | 21.6 | 21.6 | 21.6 | 43.2 | 21.6 | 0 | 1 | 17 |
| 2 | 0 | 30.8 | 23.1 | 30.8 | 7.7 | 1 | 0 | 4.4 |
| 2 | 54.6 | 72.8 | 54.6 | 145.6 | 18.2 | 1 | 1 | 9.1 |
| 2 | 0 | 42.8 | 32.1 | 85.6 | 10.7 | 1 | 0 | 8.4 |
| 2 | 6 | 6 | 6 | 6 | 6 | 0 | 1 | 4.6 |
| 2 | 114.9 | 153.2 | 114.9 | 306.4 | 38.3 | 1 | 1 | 11.5 |
| 2 | 0 | 8 | 8 | 8 | 8 | 0 | 0 | 6.7 |
| 2 | 34.8 | 46.4 | 34.8 | 92.8 | 11.6 | 1 | 1 | 10.2 |
| 2 | 11.9 | 11.9 | 11.9 | 23.8 | 11.9 | 0 | 1 | 7.9 |
| 2 | 41.4 | 55.2 | 41.4 | 55.2 | 13.8 | 1 | 1 | 2.2 |
| 2 | 0 | 13.3 | 13.3 | 26.6 | 13.3 | 0 | 0 | 7.7 |
| 2 | 0 | 66.4 | 49.8 | 66.4 | 16.6 | 1 | 0 | 5.1 |
| 2 | 19.6 | 19.6 | 19.6 | 39.2 | 19.6 | 0 | 1 | 7.1 |
| 2 | 0 | 12.4 | 12.4 | 24.8 | 12.4 | 0 | 0 | 7.3 |
| 2 | 36.8 | 36.8 | 36.8 | 73.6 | 36.8 | 0 | 1 | 26 |
| 2 | 0 | 11.9 | 11.9 | 23.8 | 11.9 | 0 | 0 | 13.9 |
| 2 | 0 | 17.1 | 17.1 | 17.1 | 17.1 | 0 | 0 | 5.6 |
| 2 | 0 | 15.5 | 15.5 | 31 | 15.5 | 0 | 0 | 7.7 |
| 2 | 13.6 | 13.6 | 13.6 | 27.2 | 13.6 | 0 | 1 | 14.2 |
| 2 | 27.9 | 37.2 | 27.9 | 37.2 | 9.3 | 1 | 1 | 4.3 |
| 2 | 144.6 | 144.6 | 144.6 | 289.2 | 144.6 | 0 | 1 | 10.3 |
| 2 | 0 | 20.4 | 20.4 | 40.8 | 20.4 | 0 | 0 | 18.8 |
| 2 | 121.8 | 162.4 | 121.8 | 324.8 | 40.6 | 1 | 1 | 10.7 |
| 2 | 24 | 32 | 24 | 32 | 8 | 0 | 2 | 6.6 |
| 2 | 410.4 | 547.2 | 410.4 | 1094.4 | 136.8 | 1 | 1 | 13.8 |
| 2 | 13.2 | 13.2 | 13.2 | 13.2 | 13.2 | 0 | 1 | 5.4 |
| 2 | 369.6 | 492.8 | 369.6 | 985.6 | 123.2 | 1 | 1 | 8.7 |
| 2 | 128.8 | 128.8 | 128.8 | 257.6 | 128.8 | 0 | 1 | 8 |
| 2 | 0 | 47.6 | 35.7 | 47.6 | 11.9 | 1 | 0 | 4 |
| 2 | 0 | 35.52 | 26.64 | 35.52 | 8.88 | 1 | 0 | 4.4 |
| 2 | 0 | 26.4 | 19.8 | 52.8 | 6.6 | 1 | 0 | 13.8 |
| 2 | 103.5 | 184 | 103.5 | 368 | 11.5 | 1 | 2 | 10.8 |
| 2 | 14.7 | 14.7 | 14.7 | 29.4 | 14.7 | 0 | 1 | 8.4 |
| 2 | 27.9 | 37.2 | 27.9 | 37.2 | 9.3 | 0 | 2 | 3.9 |
| 2 | 40.8 | 54.4 | 40.8 | 108.8 | 13.6 | 1 | 1 | 19.2 |
| 2 | 0 | 32.2 | 32.2 | 64.4 | 32.2 | 0 | 0 | 8.1 |
| 2 | 20 | 20 | 20 | 40 | 20 | 0 | 1 | 9.1 |
| 2 | 21.9 | 29.2 | 21.9 | 58.4 | 7.3 | 1 | 1 | 9.1 |
| 2 | 28.1 | 28.1 | 28.1 | 28.1 | 28.1 | 0 | 1 | 6.4 |
| 2 | 0 | 50 | 37.5 | 50 | 12.5 | 1 | 0 | 5.4 |
| 2 | 45.6 | 60.8 | 45.6 | 121.6 | 15.2 | 1 | 1 | 7.1 |
| 2 | 0 | 24.2 | 24.2 | 48.4 | 24.2 | 0 | 0 | 9.1 |
| 2 | 0 | 36 | 27 | 36 | 9 | 1 | 0 | 5 |
| 2 | 0 | 21.4 | 21.4 | 42.8 | 21.4 | 0 | 0 | 13.4 |
| 2 | 0 | 10.4 | 10.4 | 10.4 | 10.4 | 0 | 0 | 5.8 |
| 2 | 12.7 | 12.7 | 12.7 | 25.4 | 12.7 | 0 | 1 | 7.9 |
| 2 | 22.2 | 22.2 | 22.2 | 22.2 | 22.2 | 0 | 1 | 5.1 |
| 2 | 76.8 | 102.4 | 76.8 | 204.8 | 25.6 | 0 | 2 | 8.4 |
| 2 | 0 | 8.4 | 8.4 | 8.4 | 8.4 | 0 | 0 | 4.5 |
| 2 | 0 | 17.8 | 17.8 | 17.8 | 17.8 | 0 | 0 | 6.9 |
| 2 | 0 | 10.8 | 10.8 | 21.6 | 10.8 | 0 | 0 | 7.3 |
| 2 | 60 | 80 | 60 | 160 | 20 | 0 | 2 | 13.9 |
| 2 | 28.2 | 37.6 | 28.2 | 75.2 | 9.4 | 1 | 1 | 7.6 |
| 2 | 0 | 17.3 | 17.3 | 17.3 | 17.3 | 0 | 0 | 3.9 |
| 2 | 25.2 | 33.6 | 25.2 | 33.6 | 8.4 | 1 | 1 | 2.3 |
| 2 | 30.9 | 41.2 | 30.9 | 41.2 | 10.3 | 1 | 1 | 4.2 |
| 2 | 0 | 34.4 | 25.8 | 34.4 | 8.6 | 1 | 0 | 3.1 |
| 2 | 21.8 | 21.8 | 21.8 | 43.6 | 21.8 | 0 | 1 | 12.7 |
| 2 | 0 | 31.2 | 23.4 | 62.4 | 7.8 | 1 | 0 | 7.3 |
| 2 | 72 | 72 | 72 | 72 | 72 | 0 | 1 | 4.2 |
| 2 | 0 | 7.2 | 7.2 | 14.4 | 7.2 | 0 | 0 | 8.2 |
| 2 | 37.1 | 37.1 | 37.1 | 74.2 | 37.1 | 0 | 1 | 22.7 |
| 2 | 179.7 | 239.6 | 179.7 | 239.6 | 59.9 | 0 | 2 | 6 |
| 2 | 5.45 | 5.45 | 5.45 | 10.9 | 5.45 | 0 | 1 | 11.5 |
| 2 | 0 | 20.1 | 20.1 | 40.2 | 20.1 | 0 | 0 | 8.2 |
| 2 | 14.7 | 19.6 | 14.7 | 19.6 | 4.9 | 1 | 1 | 5.8 |
| 2 | 31.1 | 31.1 | 31.1 | 62.2 | 31.1 | 0 | 1 | 11.1 |
| 2 | 14.1 | 14.1 | 14.1 | 28.2 | 14.1 | 0 | 1 | 14 |
| 2 | 293.7 | 391.6 | 293.7 | 391.6 | 97.9 | 1 | 1 | 5.7 |
| 2 | 27.3 | 27.3 | 27.3 | 27.3 | 27.3 | 0 | 1 | 4.7 |
| 2 | 8.7 | 8.7 | 8.7 | 17.4 | 8.7 | 0 | 1 | 8.7 |
| 2 | 20.3 | 20.3 | 20.3 | 20.3 | 20.3 | 0 | 1 | 5.2 |
| 2 | 13.3 | 13.3 | 13.3 | 26.6 | 13.3 | 0 | 1 | 10.4 |
| 2 | 26.4 | 35.2 | 26.4 | 35.2 | 8.8 | 1 | 1 | 5.7 |
| 2 | 20.3 | 20.3 | 20.3 | 20.3 | 20.3 | 0 | 1 | 4.4 |
| 2 | 78.8 | 78.8 | 78.8 | 157.6 | 78.8 | 0 | 1 | 11.6 |
| 2 | 134.1 | 238.4 | 134.1 | 476.8 | 14.9 | 1 | 2 | 11.8 |
| 2 | 39.3 | 52.4 | 39.3 | 52.4 | 13.1 | 1 | 1 | 4.8 |
| 2 | 85.2 | 113.6 | 85.2 | 227.2 | 28.4 | 0 | 2 | 28 |
| 2 | 14.5 | 14.5 | 14.5 | 29 | 14.5 | 0 | 1 | 12.6 |
| 2 | 44.1 | 58.8 | 44.1 | 58.8 | 14.7 | 1 | 1 | 4.9 |
| 2 | 9 | 9 | 9 | 9 | 9 | 0 | 1 | 2.7 |
| 2 | 0 | 17.8 | 17.8 | 17.8 | 17.8 | 0 | 0 | 6.2 |
| 2 | 14.3 | 14.3 | 14.3 | 28.6 | 14.3 | 0 | 1 | 7 |
| 2 | 33.5 | 33.5 | 33.5 | 67 | 33.5 | 0 | 1 | 26.2 |
| 2 | 41.4 | 55.2 | 41.4 | 110.4 | 13.8 | 1 | 1 | 12 |
| 2 | 0 | 83.2 | 62.4 | 166.4 | 20.8 | 1 | 0 | 14.7 |
| 2 | 0 | 28.5 | 28.5 | 57 | 28.5 | 0 | 0 | 7 |
| 2 | 28.9 | 28.9 | 28.9 | 57.8 | 28.9 | 0 | 1 | 9.6 |
| 2 | 62.1 | 82.8 | 62.1 | 165.6 | 20.7 | 1 | 1 | 7.8 |
| 2 | 47.4 | 63.2 | 47.4 | 126.4 | 15.8 | 1 | 1 | 8.4 |
| 2 | 0 | 9.9 | 9.9 | 19.8 | 9.9 | 0 | 0 | 10 |
| 2 | 0 | 41.2 | 41.2 | 41.2 | 41.2 | 0 | 0 | 4.2 |
| 2 | 105.7 | 105.7 | 105.7 | 105.7 | 105.7 | 0 | 1 | 5.4 |
| 2 | 18.9 | 18.9 | 18.9 | 37.8 | 18.9 | 0 | 1 | 8.5 |
| 2 | 14.3 | 14.3 | 14.3 | 28.6 | 14.3 | 0 | 1 | 7.8 |
| 2 | 32.7 | 43.6 | 32.7 | 43.6 | 10.9 | 1 | 1 | 6.5 |
| 2 | 0 | 61 | 61 | 122 | 61 | 0 | 0 | 7.7 |
| 2 | 110.5 | 110.5 | 110.5 | 110.5 | 110.5 | 0 | 1 | 5 |
| 2 | 0 | 7.2 | 7.2 | 14.4 | 7.2 | 0 | 0 | 8.3 |
| 2 | 26.4 | 26.4 | 26.4 | 26.4 | 26.4 | 0 | 1 | 4.3 |
| 2 | 6.5 | 6.5 | 6.5 | 13 | 6.5 | 0 | 1 | 8.1 |
| 2 | 15.9 | 15.9 | 15.9 | 31.8 | 15.9 | 0 | 1 | 7 |
| 2 | 0 | 10.1 | 10.1 | 20.2 | 10.1 | 0 | 0 | 7.9 |
| 2 | 43.1 | 43.1 | 43.1 | 43.1 | 43.1 | 0 | 1 | 4.6 |
| 2 | 9.8 | 9.8 | 9.8 | 9.8 | 9.8 | 0 | 1 | 6.9 |
| 2 | 0 | 106 | 106 | 106 | 106 | 0 | 0 | 5.3 |
| 2 | 28.8 | 38.4 | 28.8 | 38.4 | 9.6 | 0 | 3 | 4.4 |
| 2 | 4.9 | 4.9 | 4.9 | 9.8 | 4.9 | 0 | 1 | 7.6 |
| 2 | 0 | 117.2 | 117.2 | 234.4 | 117.2 | 0 | 0 | 8.4 |
| 2 | 79.2 | 105.6 | 79.2 | 211.2 | 26.4 | 0 | 2 | 11.2 |
| 2 | 0 | 126.6 | 126.6 | 126.6 | 126.6 | 0 | 0 | 3.4 |
| 2 | 79.2 | 79.2 | 79.2 | 158.4 | 79.2 | 0 | 1 | 7.5 |
| 2 | 8.5 | 8.5 | 8.5 | 8.5 | 8.5 | 0 | 1 | 4.2 |
| 2 | 9.8 | 9.8 | 9.8 | 9.8 | 9.8 | 0 | 1 | 5.6 |
| 2 | 0 | 84.8 | 84.8 | 84.8 | 84.8 | 0 | 0 | 3.7 |
| 2 | 77.5 | 77.5 | 77.5 | 155 | 77.5 | 0 | 1 | 7.5 |
| 2 | 0 | 158.1 | 158.1 | 316.2 | 158.1 | 0 | 0 | 8.8 |
| 2 | 7.4 | 7.4 | 7.4 | 7.4 | 7.4 | 0 | 1 | 4.7 |
| 2 | 72.3 | 96.4 | 72.3 | 96.4 | 24.1 | 0 | 3 | 6.4 |
| 2 | 48.6 | 64.8 | 48.6 | 64.8 | 16.2 | 1 | 1 | 3.5 |
| 2 | 0 | 14.5 | 14.5 | 14.5 | 14.5 | 0 | 0 | 6.9 |
| 2 | 0 | 39.4 | 39.4 | 39.4 | 39.4 | 0 | 0 | 5.1 |
| 2 | 10 | 10 | 10 | 20 | 10 | 0 | 1 | 8.1 |
| 2 | 116.1 | 154.8 | 116.1 | 309.6 | 38.7 | 0 | 2 | 12.3 |
| 2 | 0 | 46.5 | 46.5 | 46.5 | 46.5 | 0 | 0 | 6.1 |
| 2 | 62.7 | 83.6 | 62.7 | 83.6 | 20.9 | 0 | 2 | 4.4 |
| 2 | 0 | 118.3 | 118.3 | 118.3 | 118.3 | 0 | 0 | 5.7 |
| 2 | 113.7 | 151.6 | 113.7 | 151.6 | 37.9 | 0 | 2 | 4.5 |
| 2 | 69.3 | 92.4 | 69.3 | 92.4 | 23.1 | 0 | 2 | 5 |
| 2 | 0 | 8.1 | 8.1 | 16.2 | 8.1 | 0 | 0 | 8.9 |
| 2 | 249.3 | 332.4 | 249.3 | 332.4 | 83.1 | 0 | 2 | 4.3 |
| 2 | 7.6 | 7.6 | 7.6 | 15.2 | 7.6 | 0 | 1 | 9.2 |
| 2 | 0 | 70.3 | 70.3 | 70.3 | 70.3 | 0 | 0 | 5 |
| 2 | 30.9 | 41.2 | 30.9 | 82.4 | 10.3 | 1 | 1 | 9.6 |
| 2 | 36.3 | 36.3 | 36.3 | 36.3 | 36.3 | 0 | 1 | 4.7 |
| 2 | 0 | 9.3 | 9.3 | 18.6 | 9.3 | 0 | 0 | 9.8 |
| 2 | 0 | 19.5 | 19.5 | 39 | 19.5 | 0 | 0 | 8.3 |
| 2 | 0 | 22 | 16.5 | 44 | 5.5 | 1 | 0 | 11.1 |
| 2 | 36.3 | 36.3 | 36.3 | 72.6 | 36.3 | 0 | 1 | 7.2 |
| 2 | 34.3 | 34.3 | 34.3 | 34.3 | 34.3 | 0 | 1 | 6.9 |
| 2 | 0 | 48 | 48 | 96 | 48 | 0 | 0 | 7.5 |
| 2 | 21 | 21 | 21 | 21 | 21 | 0 | 1 | 4.8 |
| 2 | 20.7 | 20.7 | 20.7 | 20.7 | 20.7 | 0 | 1 | 5.6 |
| 2 | 0 | 102.6 | 102.6 | 102.6 | 102.6 | 0 | 0 | 5.7 |
| 2 | 10.4 | 10.4 | 10.4 | 10.4 | 10.4 | 0 | 1 | 2.4 |
| 2 | 0 | 18.5 | 18.5 | 18.5 | 18.5 | 0 | 0 | 5.8 |
| 2 | 0 | 9.4 | 9.4 | 18.8 | 9.4 | 0 | 0 | 7 |
| 2 | 21.7 | 21.7 | 21.7 | 43.4 | 21.7 | 0 | 1 | 15.3 |
| 2 | 25.3 | 25.3 | 25.3 | 25.3 | 25.3 | 0 | 1 | 6 |
| 2 | 0 | 12.3 | 12.3 | 12.3 | 12.3 | 0 | 0 | 4.7 |
| 2 | 0 | 20.4 | 20.4 | 20.4 | 20.4 | 0 | 0 | 5.6 |
| 2 | 0 | 24.8 | 18.6 | 24.8 | 6.2 | 1 | 0 | 4.6 |
| 2 | 41.6 | 41.6 | 41.6 | 83.2 | 41.6 | 0 | 1 | 17 |
| 2 | 30.3 | 40.4 | 30.3 | 80.8 | 10.1 | 1 | 1 | 9.2 |
| 2 | 0 | 17.1 | 17.1 | 34.2 | 17.1 | 0 | 0 | 7.5 |
| 2 | 0 | 36.4 | 36.4 | 36.4 | 36.4 | 0 | 0 | 6 |
| 2 | 94.2 | 125.6 | 94.2 | 125.6 | 31.4 | 0 | 2 | 6.1 |
| 2 | 9.3 | 9.3 | 9.3 | 9.3 | 9.3 | 0 | 1 | 5.2 |
| 2 | 16.6 | 16.6 | 16.6 | 33.2 | 16.6 | 0 | 1 | 7.1 |
| 2 | 0 | 20.5 | 20.5 | 20.5 | 20.5 | 0 | 0 | 2.2 |
| 2 | 11.2 | 11.2 | 11.2 | 11.2 | 11.2 | 0 | 1 | 6.7 |
| 2 | 0 | 1000 | 1000 | 1000 | 2000 | 0 | 0 | 11.4 |
| 2 | 0 | 18.2 | 18.2 | 18.2 | 18.2 | 0 | 0 | 4.4 |
| 2 | 10.7 | 10.7 | 10.7 | 10.7 | 10.7 | 0 | 1 | 4 |
| 2 | 170.4 | 227.2 | 170.4 | 454.4 | 56.8 | 0 | 2 | 7.7 |
| 2 | 50.4 | 50.4 | 50.4 | 50.4 | 50.4 | 0 | 1 | 5.3 |
| 2 | 0 | 27 | 27 | 27 | 27 | 0 | 0 | 6.4 |
| 2 | 0 | 29.6 | 29.6 | 29.6 | 29.6 | 0 | 0 | 4.2 |
| 2 | 0 | 13.2 | 13.2 | 13.2 | 13.2 | 0 | 0 | 5.1 |
| 2 | 0 | 29.6 | 29.6 | 29.6 | 29.6 | 0 | 0 | 4.9 |
| 2 | 0 | 80.7 | 80.7 | 80.7 | 80.7 | 0 | 0 | 5.9 |
| 2 | 0 | 27.6 | 27.6 | 55.2 | 27.6 | 0 | 0 | 7.4 |
| 2 | 5.5 | 5.5 | 5.5 | 5.5 | 5.5 | 0 | 1 | 6.3 |
| 2 | 27.9 | 27.9 | 27.9 | 27.9 | 27.9 | 0 | 1 | 2 |
| 2 | 9.7 | 9.7 | 9.7 | 19.4 | 9.7 | 0 | 1 | 7.7 |
| 2 | 19.2 | 25.6 | 19.2 | 25.6 | 6.4 | 1 | 1 | 6.9 |
| 2 | 11.4 | 11.4 | 11.4 | 22.8 | 11.4 | 0 | 1 | 7.7 |
| 2 | 0 | 27.6 | 27.6 | 27.6 | 27.6 | 0 | 0 | 4 |
| 2 | 12.3 | 12.3 | 12.3 | 12.3 | 12.3 | 0 | 1 | 5.3 |
| 2 | 37.2 | 49.6 | 37.2 | 49.6 | 12.4 | 0 | 2 | 5.7 |
| 2 | 41.7 | 41.7 | 41.7 | 83.4 | 41.7 | 0 | 1 | 20.2 |
| 2 | 0 | 29.2 | 29.2 | 29.2 | 29.2 | 0 | 0 | 6.9 |
| 2 | 0 | 24.8 | 24.8 | 24.8 | 24.8 | 0 | 0 | 5.7 |
| 2 | 12.2 | 12.2 | 12.2 | 24.4 | 12.2 | 0 | 1 | 7.2 |
| 2 | 32.3 | 32.3 | 32.3 | 32.3 | 32.3 | 0 | 1 | 6 |
| 2 | 33.2 | 33.2 | 33.2 | 66.4 | 33.2 | 0 | 1 | 10.3 |
| 2 | 17.2 | 17.2 | 17.2 | 17.2 | 17.2 | 0 | 1 | 6.9 |
| 2 | 0 | 572.6 | 572.6 | 1145.2 | 572.6 | 0 | 0 | 8.2 |
| 2 | 0 | 15.9 | 15.9 | 31.8 | 15.9 | 0 | 0 | 7.7 |
| 2 | 23.2 | 23.2 | 23.2 | 23.2 | 23.2 | 0 | 1 | 5.1 |
| 2 | 8.4 | 8.4 | 8.4 | 8.4 | 8.4 | 0 | 1 | 6.9 |
| 2 | 0 | 8.1 | 8.1 | 16.2 | 8.1 | 0 | 0 | 10.2 |
| 2 | 33.6 | 33.6 | 33.6 | 67.2 | 33.6 | 0 | 1 | 7.5 |
| 2 | 16.6 | 16.6 | 16.6 | 16.6 | 16.6 | 0 | 1 | 4.7 |
| 2 | 59.4 | 79.2 | 59.4 | 79.2 | 19.8 | 1 | 1 | 5.2 |
| 2 | 0 | 21.8 | 21.8 | 43.6 | 21.8 | 0 | 0 | 7.2 |
| 2 | 0 | 9.6 | 9.6 | 9.6 | 9.6 | 0 | 0 | 4 |
| 2 | 12.6 | 12.6 | 12.6 | 12.6 | 12.6 | 0 | 1 | 4.1 |
| 2 | 12.8 | 12.8 | 12.8 | 12.8 | 12.8 | 0 | 1 | 4.1 |
| 2 | 178.6 | 178.6 | 178.6 | 357.2 | 178.6 | 0 | 1 | 31.3 |
| 2 | 12.2 | 12.2 | 12.2 | 24.4 | 12.2 | 0 | 1 | 11.7 |
| 2 | 0 | 28.2 | 28.2 | 28.2 | 28.2 | 0 | 0 | 6.6 |
| 2 | 0 | 27.6 | 27.6 | 27.6 | 27.6 | 0 | 0 | 4.4 |
| 2 | 23.5 | 23.5 | 23.5 | 47 | 23.5 | 0 | 1 | 11.5 |
| 2 | 0 | 99.4 | 99.4 | 198.8 | 99.4 | 0 | 0 | 7.3 |
| 2 | 12.1 | 12.1 | 12.1 | 12.1 | 12.1 | 0 | 1 | 3.7 |
| 2 | 0 | 49.3 | 49.3 | 49.3 | 49.3 | 0 | 0 | 3.6 |
| 2 | 0 | 9.2 | 9.2 | 9.2 | 9.2 | 0 | 0 | 4.9 |
| 2 | 11.7 | 11.7 | 11.7 | 23.4 | 11.7 | 0 | 1 | 7.7 |
| 2 | 0 | 22.6 | 22.6 | 22.6 | 22.6 | 0 | 0 | 5.4 |
| 2 | 0 | 27.6 | 20.7 | 55.2 | 6.9 | 1 | 0 | 21.5 |
| 2 | 0 | 4.8 | 4.8 | 4.8 | 4.8 | 0 | 0 | 5.5 |
| 2 | 45.6 | 45.6 | 45.6 | 91.2 | 45.6 | 0 | 1 | 10.8 |
| 2 | 62.4 | 62.4 | 62.4 | 62.4 | 62.4 | 0 | 1 | 6.2 |
| 2 | 0 | 15.2 | 15.2 | 15.2 | 15.2 | 0 | 0 | 3.9 |
| 2 | 18.1 | 18.1 | 18.1 | 18.1 | 18.1 | 0 | 1 | 6 |
| 2 | 20.1 | 20.1 | 20.1 | 20.1 | 20.1 | 0 | 1 | 6 |
| 2 | 107.7 | 107.7 | 107.7 | 107.7 | 107.7 | 0 | 1 | 3.8 |
| 2 | 37.6 | 37.6 | 37.6 | 37.6 | 37.6 | 0 | 1 | 4.2 |
| 2 | 0 | 15.9 | 15.9 | 31.8 | 15.9 | 0 | 0 | 9.7 |
| 2 | 0 | 73.3 | 73.3 | 73.3 | 73.3 | 0 | 0 | 6 |
| 2 | 11.6 | 11.6 | 11.6 | 11.6 | 11.6 | 0 | 1 | 5.8 |
| 2 | 0 | 960.7 | 960.7 | 960.7 | 960.7 | 0 | 0 | 5.8 |
| 2 | 22.5 | 22.5 | 22.5 | 22.5 | 22.5 | 0 | 1 | 4.5 |
| 2 | 56.3 | 56.3 | 56.3 | 56.3 | 56.3 | 0 | 1 | 3.8 |
| 2 | 57 | 57 | 57 | 57 | 57 | 0 | 1 | 6.7 |
| 2 | 59 | 59 | 59 | 59 | 59 | 0 | 1 | 4.7 |
| 2 | 0 | 14.2 | 14.2 | 28.4 | 14.2 | 0 | 0 | 10.3 |
| 2 | 21.2 | 21.2 | 21.2 | 21.2 | 21.2 | 0 | 1 | 5.5 |
| 2 | 573.3 | 764.4 | 573.3 | 764.4 | 191.1 | 0 | 2 | 6 |
| 2 | 16.2 | 16.2 | 16.2 | 16.2 | 16.2 | 0 | 1 | 5.2 |
| 2 | 0 | 13.4 | 13.4 | 26.8 | 13.4 | 0 | 0 | 8.8 |
| 2 | 0 | 32.8 | 24.6 | 32.8 | 8.2 | 1 | 0 | 6.3 |
| 2 | 10.1 | 10.1 | 10.1 | 10.1 | 10.1 | 0 | 1 | 5.1 |
| 2 | 50.4 | 67.2 | 50.4 | 134.4 | 16.8 | 0 | 2 | 13.5 |
| 2 | 0 | 17.7 | 17.7 | 35.4 | 17.7 | 0 | 0 | 8.6 |
| 2 | 32.2 | 32.2 | 32.2 | 32.2 | 32.2 | 0 | 1 | 5.3 |
| 2 | 0 | 11.5 | 11.5 | 11.5 | 11.5 | 0 | 0 | 4.6 |
| 2 | 14.9 | 14.9 | 14.9 | 14.9 | 14.9 | 0 | 1 | 5.2 |
| 2 | 0 | 258.3 | 258.3 | 258.3 | 258.3 | 0 | 0 | 5.1 |
| 2 | 51 | 68 | 51 | 136 | 17 | 0 | 2 | 8.8 |
| 2 | 26.7 | 35.6 | 26.7 | 35.6 | 8.9 | 1 | 1 | 4.7 |
| 2 | 0 | 28 | 28 | 56 | 28 | 0 | 0 | 7.9 |
| 2 | 23.7 | 31.6 | 23.7 | 63.2 | 7.9 | 1 | 1 | 8.7 |
| 2 | 0 | 28.9 | 28.9 | 28.9 | 28.9 | 0 | 0 | 6.8 |
| 2 | 190.8 | 254.4 | 190.8 | 508.8 | 63.6 | 0 | 2 | 11.4 |
| 2 | 172.8 | 307.2 | 172.8 | 614.4 | 19.2 | 1 | 2 | 15.4 |
| 2 | 0 | 208.6 | 208.6 | 208.6 | 208.6 | 0 | 0 | 6.8 |
| 2 | 0 | 8.4 | 8.4 | 8.4 | 8.4 | 0 | 0 | 5.2 |
| 2 | 0 | 21.2 | 21.2 | 21.2 | 21.2 | 0 | 0 | 4.9 |
| 2 | 0 | 115.3 | 115.3 | 115.3 | 115.3 | 0 | 0 | 5.1 |
| 2 | 0 | 52.2 | 52.2 | 52.2 | 52.2 | 0 | 0 | 4.4 |
| 2 | 24.2 | 24.2 | 24.2 | 24.2 | 24.2 | 0 | 1 | 5.5 |
| 2 | 0 | 52.5 | 52.5 | 52.5 | 52.5 | 0 | 0 | 6 |
| 2 | 0 | 163.7 | 163.7 | 327.4 | 163.7 | 0 | 0 | 7.2 |
| 2 | 0 | 20.5 | 20.5 | 20.5 | 20.5 | 0 | 0 | 5.8 |
| 2 | 103.8 | 138.4 | 103.8 | 276.8 | 34.6 | 0 | 2 | 8.4 |
| 2 | 0 | 94.7 | 94.7 | 94.7 | 94.7 | 0 | 0 | 4.8 |
| 2 | 23.9 | 23.9 | 23.9 | 23.9 | 23.9 | 0 | 1 | 6.3 |
| 2 | 0 | 13.4 | 13.4 | 26.8 | 13.4 | 0 | 0 | 8.2 |
| 2 | 30.6 | 40.8 | 30.6 | 81.6 | 10.2 | 1 | 1 | 9.5 |
| 2 | 0 | 16.7 | 16.7 | 16.7 | 16.7 | 0 | 0 | 3.8 |
| 2 | 13.8 | 13.8 | 13.8 | 13.8 | 13.8 | 0 | 1 | 5.2 |
| 2 | 13.2 | 13.2 | 13.2 | 13.2 | 13.2 | 0 | 1 | 4.8 |
| 2 | 50.7 | 67.6 | 50.7 | 67.6 | 16.9 | 0 | 2 | 6.7 |
| 2 | 0 | 21 | 21 | 21 | 21 | 0 | 0 | 6.3 |
| 2 | 0 | 40.5 | 40.5 | 40.5 | 40.5 | 0 | 0 | 4.7 |
| 2 | 0 | 29.6 | 29.6 | 29.6 | 29.6 | 0 | 0 | 4.4 |
| 2 | 0 | 11.4 | 11.4 | 11.4 | 11.4 | 0 | 0 | 5.2 |
| 2 | 0 | 107.4 | 107.4 | 107.4 | 107.4 | 0 | 0 | 6.3 |
| 2 | 0 | 175.6 | 175.6 | 175.6 | 175.6 | 0 | 0 | 5.7 |
| 2 | 151.4 | 151.4 | 151.4 | 151.4 | 151.4 | 0 | 1 | 5.2 |
| 2 | 138.9 | 185.2 | 138.9 | 370.4 | 46.3 | 1 | 1 | 10.4 |
| 2 | 0 | 21 | 21 | 42 | 21 | 0 | 0 | 7.6 |
| 2 | 48.7 | 48.7 | 48.7 | 97.4 | 48.7 | 0 | 1 | 16.3 |
| 2 | 33.88 | 33.88 | 33.88 | 33.88 | 33.88 | 0 | 1 | 5.4 |
| 2 | 34.2 | 45.6 | 34.2 | 45.6 | 11.4 | 1 | 1 | 6.3 |
| 2 | 0 | 52.3 | 52.3 | 52.3 | 52.3 | 0 | 0 | 6.9 |
| 2 | 69.8 | 69.8 | 69.8 | 139.6 | 69.8 | 0 | 1 | 7.2 |
| 2 | 0 | 8.7 | 8.7 | 8.7 | 8.7 | 0 | 0 | 2.9 |
| 2 | 388.8 | 518.4 | 388.8 | 518.4 | 129.6 | 0 | 2 | 6.5 |
| 2 | 28 | 28 | 28 | 56 | 28 | 0 | 1 | 7.8 |
| 2 | 0 | 42 | 42 | 84 | 42 | 0 | 0 | 7.1 |
| 2 | 0 | 34.3 | 34.3 | 34.3 | 34.3 | 0 | 0 | 6.6 |
| 2 | 18.2 | 18.2 | 18.2 | 18.2 | 18.2 | 0 | 1 | 5.6 |
| 2 | 63.9 | 85.2 | 63.9 | 170.4 | 21.3 | 0 | 2 | 8.4 |
| 2 | 36.1 | 36.1 | 36.1 | 72.2 | 36.1 | 0 | 1 | 11.8 |
| 2 | 0 | 37.1 | 37.1 | 37.1 | 37.1 | 0 | 0 | 5 |
| 2 | 0 | 21.9 | 21.9 | 21.9 | 21.9 | 0 | 0 | 5.7 |
| 2 | 14.1 | 14.1 | 14.1 | 28.2 | 14.1 | 0 | 1 | 8.1 |
| 2 | 0 | 24 | 24 | 24 | 24 | 0 | 0 | 4.6 |
| 2 | 0 | 51.6 | 51.6 | 51.6 | 51.6 | 0 | 0 | 6.3 |
| 2 | 0 | 52.3 | 52.3 | 104.6 | 52.3 | 0 | 0 | 9 |
| 2 | 26 | 26 | 26 | 52 | 26 | 0 | 1 | 12.6 |
| 2 | 0 | 24.8 | 18.6 | 49.6 | 6.2 | 1 | 0 | 17 |
| 2 | 187.6 | 187.6 | 187.6 | 187.6 | 187.6 | 0 | 1 | 5.1 |
| 2 | 24.7 | 24.7 | 24.7 | 24.7 | 24.7 | 0 | 1 | 4.3 |
| 2 | 13 | 13 | 13 | 13 | 13 | 0 | 1 | 3.3 |
| 2 | 0 | 12.3 | 12.3 | 12.3 | 12.3 | 0 | 0 | 5.2 |
| 2 | 13.1 | 13.1 | 13.1 | 13.1 | 13.1 | 0 | 1 | 6.7 |
| 2 | 35.1 | 46.8 | 35.1 | 93.6 | 11.7 | 1 | 1 | 12.6 |
| 2 | 157.5 | 280 | 157.5 | 280 | 17.5 | 1 | 2 | 2.3 |
| 2 | 0 | 24 | 24 | 24 | 24 | 0 | 0 | 3.2 |
| 2 | 33.6 | 33.6 | 33.6 | 67.2 | 33.6 | 0 | 1 | 8.7 |
| 2 | 0 | 59.9 | 59.9 | 119.8 | 59.9 | 0 | 0 | 8 |
| 2 | 11 | 11 | 11 | 11 | 11 | 0 | 1 | 5.6 |
| 2 | 0 | 146.7 | 146.7 | 293.4 | 146.7 | 0 | 0 | 8 |
| 2 | 0 | 41.2 | 41.2 | 82.4 | 41.2 | 0 | 0 | 10.5 |
| 2 | 66.9 | 66.9 | 66.9 | 66.9 | 66.9 | 0 | 1 | 4 |
